# Path Learning in Individuals With Down Syndrome: The Floor Matrix Task and the Role of Individual Visuo-Spatial Measures

**DOI:** 10.3389/fnhum.2020.00107

**Published:** 2020-03-31

**Authors:** Chiara Meneghetti, Enrico Toffalini, Silvia Lanfranchi, Barbara Carretti

**Affiliations:** ^1^Department of General Psychology, University of Padua, Padua, Italy; ^2^Department of Developmental Psychology and Socialization, University of Padua, Padua, Italy

**Keywords:** down syndrome, route learning, floor Matrix, working memory, visuo-spatial abilities, environment measures

## Abstract

Environment learning is essential in everyday life. In individuals with Down syndrome (DS), this skill has begun to be examined using virtual exploration. Previous studies showed that individuals with DS can learn and remember paths in terms of sequences of turns and straight stretches, albeit with some difficulty, and this learning is supported by their cognitive abilities. This study further investigates environment learning in the DS population, newly examining their ability to learn a path from actual movements, and to learn increasingly long paths, and how their performance relates to their visuo-spatial abilities and everyday spatial activities. A group of 30 individuals with DS and 30 typically-developing (TD) children matched for receptive vocabulary performed a 4 × 4 Floor Matrix task in a grid comprising 16 squares (total area 2.3 × 2.3 meters). The task involved repeating increasingly long sequences of steps by actually moving in the grid. The sequences were presented in two learning conditions, called Observation (when participants watched the experimenter’s moves), or Map (when they were shown a map reproducing the path). Several visuo-spatial measures were also administered. The results showed a clear difference between the two groups’ performance in the individual visuo-spatial measures. In the Floor Matrix task, after controlling for visuo-spatial reasoning ability, both groups benefited to the same degree from the Observation condition vis-à-vis the Map condition, and no group differences emerged. In the group with DS, visuo-spatial abilities were more predictive of performance in the Floor Matrix task in the Observation condition than in the Map condition. The same was true of the TD group, but this difference was much less clear-cut. The visuo-spatial working memory and visualization tasks were the strongest predictors of Floor Matrix task performance. Finally, the group with DS showed a significant relation between Floor Matrix task performance in the Observation condition and everyday spatial activity. These results enlarge on what we know about path learning in individuals with DS and its relation to their visuo-spatial abilities. These findings are discussed within the frame of spatial cognition and the atypical development domain.

## Introduction

### Path Learning in Individuals With Down Syndrome

Knowing how to find your way through an environment is essential in everyday life. When people experience a new environment, they form an internal mental representation of it, showing elements (such as landmarks) and their relations, called cognitive maps (Tolman, 1948). This spatial information can be acquired using different modalities, such as from looking at maps or from navigation. Maps depict a whole area, showing landmarks and paths connecting them based on an aerial view of the layout, so they present the information in an allocentric way. Route learning by navigation involves memorizing a particular sequence of movements and changes of direction, and a set of place-action associations in order to reach a destination. In navigation the environment is experienced from an egocentric point of view, based on sensorimotor (e.g., vestibular and kinesthetic) information identifying an individual’s positions in space and self-to-object distances (Montello, 2005). The related representations and their features can be assessed using various tasks, such as map drawing or retracing a previously-explored route.

Learners’ personal characteristics are a source of variability in how successfully they retain environmental information (Meneghetti et al., 2019). When investigating the features of mental maps and the conditions that favor their formation, individuals with Down syndrome (DS) seem an interesting population to study because of the constraints imposed by the syndrome. DS is a genetic syndrome caused by chromosome 21 trisomy. Individuals with DS generally have an intelligence quotient between 25 and 70, and a mental age of between 5 and 6 years (Dykens et al., 2000; Kittler et al., 2008). It is worth further analyzing the visuo-spatial skills of individuals with DS because, although they are generally considered a relative strength (Dykens et al., 2000; Silverman, 2007), they have yet to be extensively explored. For example, these individuals’ environment learning ability (which is one type of visuo-spatial ability) has been little investigated compared with other visuo-spatial skills (see below). There is growing interest in how individuals with DS gain confidence with moving around and reaching places outside their home (a workplace, supermarket, gym, or other people’s homes), and returning home by various means (walking, taking public transport, or asking someone for directions; Yang et al., 2018). Broadening our knowledge of the DS population’s environment learning can shed light on their adaptability and capacity for autonomy, which are strongly related to their quality of life. There is reason to believe that individuals with DS can encounter difficulties with environmental learning, based on neuropsychological evidence. We know that hippocampal structures (and other related brain regions, like the parahippocampal cortex) are involved in navigation (e.g., Burgess et al., 2002). We also know that hippocampal volumes have been found smaller in individuals with DS than in matched typically-developing (TD) individuals (Pinter et al., 2001; Contestabile et al., 2010), and that the former perform less well than the latter in cognitive tasks measuring hippocampal functions (Pennington et al., 2003).

Visuo-spatial abilities have commonly been found relatively stronger than verbal abilities in individuals with DS (Dykens et al., 2000; Silverman, 2007). It would be wrong to generalize from this observation, however, since visuo-spatial abilities include a whole set of different skills. Any individual may have different strengths and weaknesses relating to a given construct, and different tasks may be used to assess these various subsets of skills. Most studies examined visuo-spatial abilities in terms of small-scale abilities, when the spatial information needing to be handled concerned objects, figures, etc., in small spaces (i.e., smaller than the individual’s body), as in paper-and-pencil tasks. A few studies examined large-scale abilities, i.e., for managing information about, or moving in larger spaces, as in the case of learning a path (Yang et al., 2014; Meneghetti et al., 2019). For instance, Yang et al. (2014)’s review showed that most of the small-scale abilities examined refer to: recalling locations (placing previously-seen objects in their appropriate positions in an empty layout, e.g., Vicari et al., 2005); closure (combining different pieces of information into larger wholes, and breaking larger wholes down into smaller parts; e.g., Vicari et al., 2006); mental rotation (mentally turning 2D and 3D objects; Hinnell and Virji-Babul, 2004; Vicari et al., 2006; Meneghetti et al., 2018); and visuo-spatial construction (reconstructing a whole object from a number of parts, as typically assessed with the WISC block design test; Cornish et al., 1999; Wechsler, 2003; Kittler et al., 2004). The results of such studies vary. For instance, they usually show that individuals with DS perform less well than TD children -matched (or controlled) for general cognitive functioning – in recalling locations and closure, while the results for mental rotation and visuo-spatial construction are less consistent. Another ability amply investigated in individuals with DS is visuo-spatial working memory (WM), which is concerned with retaining and processing visuo-spatial information, and can be distinguished as sequential or simultaneous (remembering increasingly long sequences or locating increasingly large numbers of elements, respectively, Cornoldi and Vecchi, 2003). There is evidence of individuals with DS performing as well as matched TD children in sequential WM tasks that involved remembering in the right order positions presented one at a time on a matrix. In contrast, individuals with DS performed comparatively less well in simultaneous WM tasks, which involved recalling the position of colored cells displayed simultaneously on a matrix (Lanfranchi et al., 2004, 2009; Carretti and Lanfranchi, 2010).

Only a few studies conducted to date examined large-scale visuo-spatial abilities, such as path learning, in individuals with DS, however, and most of them did so using the exploration of virtual environments (VE).

#### Path Learning in Virtual Environments

To our knowledge, there have been five studies on path learning in individuals with DS (Courbois et al., 2013; Davis et al., 2014; Farran et al., 2015; Purser et al., 2015; Toffalini et al., 2018). They all support the impression that individuals with DS are more confident when forming environment representations with egocentric features (as seen from the person’s point of view), while they have more difficulty with forming allocentric representations (based on relations between landmarks). In most of the studies, participants were asked to learn a virtual indoor or outdoor path presented from the person’s point of view. Then they were asked to reproduce the previously-seen path by trial and error until they completed it without making any mistakes, with a maximum of attempts allowable (the criterion condition). This was conceived as an egocentric task as it retained the person’s own point of view. Afterward, participants were asked to find a shortcut to their destination. This was conceived as an allocentric task as it involved linking landmarks located in the layout and taking new paths (not previously covered). It should be noted that the paths to learn generally consisted of about four segments (based on the number of turns and straight stretches), i.e., in a regular environment (a grid of 3 × 3 streets), participants needed to learn two segments for each of two paths (Courbois et al., 2013; Farran et al., 2015), or in a square-shaped environment they had to cover all four sides (Toffalini et al., 2018), or in an irregular environment they had to take a path with around four choice points (Davis et al., 2014; Purser et al., 2015).

The results of these studies show that individuals with DS were able to recall (Davis et al., 2014) or recognize landmarks irrespective of their position (Courbois et al., 2013; Toffalini et al., 2018), though their performance was not as good as that of matched TD controls. They could also trace previously-learnt paths, but it took them more attempts to do so, and they made more mistakes (Courbois et al., 2013; Davis et al., 2014; Farran et al., 2015; Purser et al., 2015), or took longer (Toffalini et al., 2018). When asked to find a shortcut, they had a clearly worse performance than TD controls (Courbois et al., 2013; Farran et al., 2015), and the few individuals with DS who succeeded in producing representations with allocentric feature still preferred to use a strategy based on their personal point of view in their moves (Farran et al., 2015). The evidence that individuals with DS can form environment representations with egocentric, but not with allocentric features supports the assumption that large-scale environment knowledge is first acquired egocentrically, and it is only afterward that individuals can form allocentric mental representations of an environment (Siegel and White, 1975).

Given that individuals with DS seem able to handle egocentric-sequential information (as expressed by their ability to repeat a previously-explored path) better than allocentric-simultaneous information, it seemed worthwhile to examine in the present study how much sequential information presented from a personal point of view they are able to learn and recall in the right order.

#### Path Learning With Actual Moves

Any actual moves are necessarily egocentric because of the body’s involvement (Montello, 2005), and they are an interesting condition to consider when examining path learning in individuals with DS.

It has been demonstrated that individuals with DS placed in a controlled setting, such as a platform about 4 m square (drawing inspiration from the Water maze task; Morris, 1984), are just as capable of making moves using distinctive local cues as matched TD children, while they perform less well when they need to use environmental information such as the locations of other elements in the layout, or the edge of a platform (Mangan, 1992; Pennington et al., 2003; Lavenex et al., 2015). Studies on the typically-developing population, designed mainly to shed light on the development of egocentric and allocentric representations, used a similarly controlled setting (like a platform) to examine children’s actual moves to reach a target (such as a hidden toy) that could be identified from local cues (e.g., colored elements) or environmental features (other elements on or off the platform). The studies showed that TD children learn to form egocentric representations by 2–5 years old, and can even use environmental information, such as the shape of a room (Hermer and Spelke, 1994; Newcombe et al., 1998). At around 6 years of age their mental representations can become view-independent, showing the children’s ability to use the structural features of an environment to infer target locations (e.g., Nardini et al., 2008, 2009; Ruggiero et al., 2016).

In the case of individuals with DS, findings obtained with tasks that involved moves in controlled settings showed that they were able to reach places using information learned from a personal point of view, and the same was true of studies using virtual exploration (Courbois et al., 2013; Davis et al., 2014; Farran et al., 2015; Purser et al., 2015). However, they had difficulty using environmental information related to a whole layout, and organizing elements in relation to each other (i.e., allocentrically), even when they should have been able to do so according to their mental age (given evidence of TD children definitely being able to using environmental information by around 6 years old).

Using a controlled real-life setting could be an interesting way to investigate to what extent individuals with DS are able to learn sequences of actual moves. This condition represents a large-scale setting in “vista space” (where the spatial setting and the movements inside it are all visible to participants; Montello, 1998). Given the current paucity of evidence concerning the ability of individuals with DS to learn sequences from actual moves in controlled settings (Mangan, 1992; Pennington et al., 2003; Lavenex et al., 2015), it is worth considering some inspiring studies conducted in the TD domain. In an effort to distinguish between small-scale and large-scale processing abilities (as used for navigation), Piccardi et al. (2008) suggested a 1:10 scaled-up version of the classical Corsi Block-Tapping Test (CBT; Corsi, 1972; a board with nine blocks irregularly placed on it), called the Walking Corsi Test (WalCT; Piccardi et al., 2008), i.e., a space marked on the floor (3 × 2.5 meters in size) with squares irregularly placed inside it. The task involved actually walking around the floor and repeating the same sequence of moves so as to pass through the same squares as an examiner had previously done (stopping in each square for 2 s). Since this task assesses the ability to learn increasingly long sequences of moves (generating a measure of span), it is conceived as a navigational WM task. TD children proved able to perform this task: from 5.4 to 6.7 years of age, which largely corresponds to the mental age of individuals with DS, they were able to reproduce sequences of up to about 3 squares (from *M* = 1.90, *SD* = 1.18 up to *M* = 2.43, *SD* = 0.84; Piccardi et al., 2014). There was evidence of their performance in the WalCT gradually improving, and becoming stable by 10 years of age, with virtually no gender-related differences (Piccardi et al., 2014). It should be noted that, when asked to learn a sequence of 4 squares, 6 years old were already able to do so, though they had more difficulty than older children, around 11 years old (Piccardi et al., 2015). The WalCT seems specifically to capture the processing ability involved in moving within a vista space setting, which differs from the processing ability involved in small-scale WM tasks. In fact, TD 5–6 years old performed less well in the WalCT than in the CBT or a verbal WM task that involved repeating increasingly long series of digits (Piccardi et al., 2014). In other words, the WalCT is a task capable of providing a key to elucidating the type of strength or weakness in participants’ navigation ability (e.g., Bianchini et al., 2010; Palermo et al., 2014).

Overall, this kind of vista space task (which is a way to reproduce a large-scale environment in a controlled setting) holds promise for assessing the path learning ability of individuals with DS. The actual moves necessarily involve adopting the egocentric view, enabling us to examine participants’ ability to learn a path based on information gained from a personal point of view.

It is important to note individuals with DS benefit from being given a regular visuo-spatial context, such as a uniform grid layout (Carretti et al., 2013), so a vista space setting with regular squares placed within a grid (as reproduced virtually by Courbois et al., 2013; Farran et al., 2015) could represent a favorable condition for assessing path learning with actual moves in the DS population.

The first aim of the present study was therefore to examine the ability of individuals with DS to learn a path from increasingly long sequences of actual moves.

### The Role of Cognitive Abilities in Supporting Path Learning

It is generally assumed that large-scale abilities (like path learning) are related to small-scale abilities (Hegarty et al., 2006), which include a large set of skills used in basic processing, such as WM, and higher-level functions like mental rotation (Hegarty and Waller, 2005). The relation between small-scale (spatial) abilities and environment learning performance has been demonstrated in adults (Hegarty et al., 2006; Weisberg et al., 2014) as well as in developmental age, in 5–6 years old children (e.g., Fenner et al., 2000; Purser et al., 2012, 2015; Merrill et al., 2016; Thomas et al., 2016), albeit with some inconsistencies in the findings. In a recent study, Merrill et al. (2016) found that the visuo-spatial abilities – i.e., mental rotation, spatial visualization (the ability to arrange spatial stimuli), and visuo-spatial working memory (in a task resembling those used to test simultaneous WM) – of children 6–12 years old were related to their path learning accuracy (after exploring VE), but it was only in females that verbal WM was also a significant predictor of their performance. Fenner et al. (2000) also found visuo-spatial abilities (including visualization, mental rotation and tasks resembling those used to test sequential WM) related to path learning (after exploring VE) in 5–6 years old, but not in 8–9 year old children. Other studies found additional cognitive abilities involved in path learning (again after exploring VE), including attention, perception, memory and executive functions (Purser et al., 2012; Nys et al., 2015). There is also evidence of 6 year old performance in the WalCT being related to individual visuo-spatial factors, such as field-independent cognitive style (Boccia et al., 2019), and even verbal abilities (such as grammar comprehension, when the squares used in the WalCT are identified with images reproducing landmarks; Piccardi et al., 2015).

It is useful to analyze the contribution of the individual (small-scale) abilities involved in environment learning (a large-scale ability) because this helps us to pinpoint factors that can explain variability in people’s performance. Analyzing these issues can be particularly important in the atypical development domain, given the more variable spatial performance of the individuals concerned (Yang et al., 2014). The few studies on individuals with DS (Davis et al., 2014; Farran et al., 2015; Purser et al., 2015; see also Lavenex et al., 2015) found a role for both visuo-spatial reasoning [measured with Raven’s colored progressive matrixes (CPM); Raven et al., 1998] and other cognitive abilities like executive control, attention and memory in participants’ environment learning (especially in path reproduction). Visuo-spatial reasoning seems to have a fundamental role in path learning, in both TD and DS groups (Farran et al., 2015), but possibly even more so in the latter (Purser et al., 2015). These findings should be considered with caution because of the small sample sizes considered, but they do seem to suggest that several cognitive abilities – and visuo-spatial reasoning in particular – are involved in supporting path learning (Farran et al., 2015; Purser et al., 2015). Other visuo-spatial abilities, such as visuo-spatial WM, visualization and mental rotation, should be considered in individuals with DS too, as studies on TD populations have found them involved in environment learning (Fenner et al., 2000; Merrill et al., 2016).

Hence the second aim of the present study, which was to examine the role of a set of visuo-spatial cognitive abilities in supporting path learning by individual with DS.

### Rationale and Aims of the Study

On the basis of the literature reviewed, the aims of the present study were to examine: a) the ability of individuals with DS to learn actual paths (in a vista space setting) of increasing length (in terms of the number of steps involved in a sequence), by comparison with matched TD children; and b) how path learning performance relates to visuo-spatial cognitive abilities and everyday spatial activities.

To address these aims, groups of individual with DS and TD children were presented with a large-scale (vista) task (inspired by Piccardi et al., 2014), in which cells were arranged in a uniform grid (a facilitating feature for individuals with DS; Carretti et al., 2013). The task was based on a span-like procedure, as used by Piccardi et al. (2014), to identify the longest sequence participants could learn (i.e., the range of their performance).

Since the way in which spatial information is presented can influence our mental representation of it (e.g., Picucci et al., 2013), two path learning conditions were presented: one involved learning from observation (Observation condition), in which participants watched a person move through a sequence of squares (as in Piccardi et al., 2014); in the other participants learned from a map (Map condition), showing the cells in the grid involved in the sequence of steps. This solution was chosen based on the evidence that children aged 3–5 years understand the representative function of maps in showing a correspondence with a larger space (e.g., Frick and Newcombe, 2012), although they have trouble with handling it flexibly (rotating it, for example; Vosmik and Presson, 2004). Preschoolers are able to use a map illustrating spatial information in a layout to address related spatial tasks involving a room (Bluestein and Acredolo, 1979; Shusterman et al., 2011) or larger spaces (Uttal and Wellman, 1989; Sandberg and Huttenlocher, 2001; Uttal et al., 2006). Uttal and Wellman (1989) found that 4 years old children shown a map indicating a path through the layout of a playhouse were better able to reproduce the path with actual moves than children not shown the map. Six years old children also proved capable of using a map at the same time as they actually moved in an environment, such as a hallway (Sandberg and Huttenlocher, 2001). These results suggest that preschoolers can integrate information shown on a map (allocentric view) while completing navigation tasks (egocentric view), in line with studies showing their ability to integrate allocentric and egocentric information (Nardini et al., 2008, 2009). This matter has been poorly investigated in individuals with DS, however, and the few studies on the effect of seeing a sketch map before learning a path through an environment found that individuals with DS did not benefit from the map in the same way as matched TD children (Toffalini et al., 2018; see also Meneghetti et al., 2017).

To address our second aim, the two groups were administered a series of visuo-spatial measures. Some assess basic processing ability, such as tasks measuring sequential and simultaneous aspects of visuo-spatial WM (i.e., the ability to manage increasingly long sequences and configurations of elements, respectively). The (small-scale) sequential WM task could be particularly relevant because the (large-scale) Floor Matrix task is based on sequences to learn, and the two types of task are related (Piccardi et al., 2014). Other measures were used to assess higher-level abilities, such as visualization and mental rotation (i.e., the ability to arrange and to rotate stimuli, respectively), that have been shown to influence path learning in TD children (Fenner et al., 2000; Merrill et al., 2016).

An everyday spatial activity questionnaire was also administered to assess the extent to which participants’ Floor Matrix task performance was associated with their performance in everyday spatial activities.

We expected to find:

(a) that both the TD and the DS group would be able to complete the Floor Matrix task with sequences involving from 2 to 4 steps. Our assumption was based on: previous evidence of TD children of an age comparable with the mental age of DS individuals being able to reproduce sequences comprising 1.90–2.44 steps correctly in an irregular vista space setting (Piccardi et al., 2014); and on VE studies on individuals with DS showing that they were able to learn paths involving 2–4 segments (including turns and straight stretches; Courbois et al., 2013; Davis et al., 2014; Farran et al., 2015; Purser et al., 2015; Toffalini et al., 2018). Differences between the groups in relation to learning condition were explored. If individuals with DS preferred the person’s point of view for acquiring spatial information (as suggested by VE studies; e.g., Courbois et al., 2013; Farran et al., 2015), and had more difficulty learning the same information presented on a map (Meneghetti et al., 2017; Toffalini et al., 2018), we might expect the group with DS to perform better in the Observation than in the Map condition. On the other hand, if TD children are able to transfer information from a map to their personal point of view (expressed by their moves in an environment; e.g., Uttal and Wellman, 1989; Sandberg and Huttenlocher, 2001), they might perform the Floor Matrix tasks just as well in the Map condition as in the Observation condition. It might be that the TD children’s performance is better in the Observation condition than in the Map condition, however. This would be due to their retaining a preference for egocentric representations (Siegel and White, 1975; Ruggiero et al., 2016);

(b) an involvement of (small-scale) visuo-spatial cognitive abilities in the performance of the Floor Matrix task in both groups, TD children and individuals with DS (Farran et al., 2015; Purser et al., 2015; Merrill et al., 2016). Differences in their involvement as a function of learning condition (Observation vs. Map) were explored in relation to the type of visuo-spatial tasks administered. Among several possibilities, visuo-spatial WM, and especially sequential WM could be involved in the Floor Matrix task, given that both rely on learning a sequence (Piccardi et al., 2015), an ability that individuals with DS also develop (Lanfranchi et al., 2004; Lanfranchi et al., 2009).

We also expected Floor Matrix task performance to be related with participants’ everyday behavior, as suggested by the evidence of a link between vista space tasks and individuals’ attitudes to moving in the environment (e.g., their self-assessed sense of direction, Mitolo et al., 2015), and their environment navigation difficulties (Palermo et al., 2014). Differences emerging between learning conditions and groups were examined in this respect.

## Materials and Methods

### Participants

A group of 30 individuals with DS (11 females; *M*_age_ = 12.72 years; *SD* = 3.44; age range = 7.75–17.92 years), and a group of 30 TD children (11 females; *M*_age_ = 5.49 years; *SD* = 0.23; age range = 5.17–6.00 years) participated in the study. The two groups were similarly distributed by gender. As the measure for matching the two groups we chose to use the Peabody Picture Vocabulary Test-Revised (PPVT-R; Dunn and Dunn, 1981; Italian adaptation by Stella et al., 2000), a measure of receptive vocabulary (aware of the complexity of the issue concerning how to match groups for age equivalence in the presence of a population characterized by peaks and troughs, e.g., Jarrold and Brock, 2004). The TD group was selected from a larger group of 90 TD children aged 5–6 years old, which can be considered as the mean equivalent age, in terms of intellectual functioning, of individuals with DS from adolescence onward (Dykens et al., 2000). This age range is also considered adequate for a child to fully understand the necessary verbal instructions and perform the Floor Matrix task. The PPVT-R consists of a series of 175 pictorial stimuli of increasing difficulty, each comprising 4 black-and-white drawings. The respondent is asked to indicate which of the four drawings best represents the word an experimenter speaks aloud when presenting each stimulus. The task is terminated when the respondent makes six mistakes in eight consecutive responses. The final score is the total number of correctly chosen drawings. The DS group had an average PPVT-R score of *M* = 67.2 (*SD* = 25.91), corresponding to a mean equivalent age of 5 years, 9 months (Stella et al., 2000). The TD group had an average score of *M* = 69.13 (*SD* = 15.62), corresponding to a mean equivalent age of 5 years, 11 months. The between-group difference was negligible, Cohen’s *d* = 0.09.

### Material

#### Floor Matrix task (Adapted From Mitolo et al., 2015)

**FIGURE 1:**
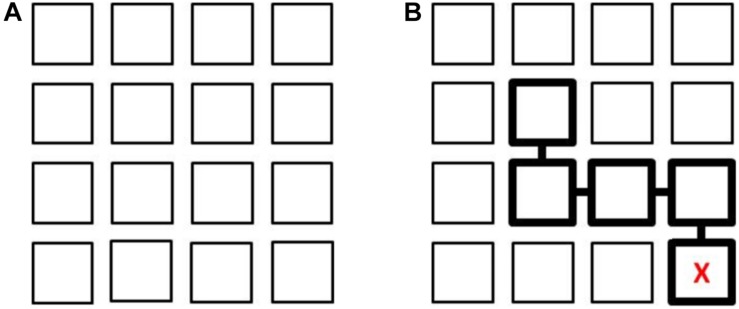
Layout of the 4 × 4 Floor Matrix task **(A)**. Example of a sequence of 4 steps with the starting point identified by a “X” **(B)**. In the Map learning condition, this is shown to the participant; in the Observation learning condition, the experimenter stands in the square with the “X” and then moves to each square in the sequence. After each learning phase, participants stand on the square marked with an “X” and reproduce the four steps in the same order.

This task assesses path learning from actual movements in a controlled vista space setting. It consists of a 4 × 4 matrix on the floor comprising 16 squares (of stiff cardboard) 50 × 50 cm in size, with a 10 cm gap between them, forming a whole square layout about covering 2.30 × 2.30 meters. The layout of the Floor Matrix task is shown in Figure 1A. The Floor Matrix is aligned with the walls of the room to avoid a mismatch between the matrix (local) and room (global) spaces, which would affect performance (Shelton and McNamara, 2001; Lavenex et al., 2015). The task involves looking at sequences of positions presented consecutively, one square at a time, on the matrix and then reproducing them in the same order. The starting position is in one of the 4 squares in the bottom row of the matrix marked with an “X” (Figure 1B). There were 14 trials (two for each number of steps in a path, which ranged from 1 to 7).

Two learning conditions were considered: in one participants watched the examiner make a series of moves to complete the sequence (Observation), while in the other they looked at a map (Map condition). In the Observation condition the experimenter stood on the square (“X” marked), then completed a sequence of steps, stopping for 3 s in each square, while a participant stood outside the matrix and watched the experimenter’s moves. The time spent on observing the sequence ranged from 3 to 21 s (for paths from 1 to 7 steps), plus the time taken to move from one square to the next (up to 1 s for each step). In the Map condition a participant was given 8 s to look at the matrix layout (a pilot study had suggested this was enough to memorize the path without participants’ attention being distracted) on a sheet of paper (16 × 16 cm) reproducing the layout on the floor, with the starting square marked with an “X” and the sequence of steps indicated by squares linked together and highlighted with thicker edges (Figure 1B). During the learning phase, in both conditions, participants stood outside the matrix in front of the square marked with an “X,” either observing the experimenter’s movements or looking at a map.

Then they were asked immediately afterward to reproduce exactly the same sequence of steps, covering the same path by walking in the matrix. To ensure that participants understood what was required of them, the experimenter first explained the task by means of verbal instructions, then provided a direct demonstration. Two trials (with 2-step sequences) were used for each condition for familiarization purposes: if participants made a mistake, the experimenter demonstrated the right move and asked them to repeat it until they could complete the sequence correctly.

The paths involved consecutive squares that could go in one of three directions (forward or to right or left). All paths were randomly chosen so as not to feature any particular regular pattern. The task was terminated when a participant failed to reproduce both trials with the same number of steps correctly. It should be noted that the sequences in the Floor Matrix task involved moving from one square to another adjacent square, whereas the sequences to recall were not adjacent in the other visuo-spatial WM tasks (e.g., the Corsi Blocks task and the sequential WM task, see below). The final score for each condition was the participant’s memory span, i.e., the maximum number of steps correctly reproduced by the participant in at least one of a pair of trials, and it ranged from 0 to 7.

#### Visuo-Spatial Reasoning

Raven’s Colored Progressive Matrices (CPM; Raven et al., 1998; Italian adaptation by Belacchi et al., 2008). This is a measure of fluid reasoning that uses items of a visuo-spatial nature. It consists of 36 increasingly complex colored matrices, and each matrix has a piece missing: the respondent is asked to choose the best fit for the missing piece from among six options. The reliability is good: the test-retest stability and convergent validity with other intelligence tests is strong in all international versions of the CPM, with *r* in 0.60–0.90 (Belacchi et al., 2008). The final score is the number of matrices correctly completed (and ranges between 0 and 36).

#### Visuo-Spatial Individual Measures

Ghost Picture Test (GPT; adapted from Frick et al., 2013). This is a measure of mental rotation ability. It consists of 21 items, each depicting a target silhouette of a ghost inside a circle on the top of the page, and two similar silhouettes underneath. The respondent has to choose which one is identical to the target figure in a rotated position (the alternative figure is a mirror image). The items require different degrees of rotation to match the target figure, i.e., 0° (3 items), 45° (3 items), 90° (7 items), 135° (4 items), and 180° (4 items). The internal consistency is good: Cronbach’s alpha calculated on the matrix of the tetrachoric correlations (because the responses are of binomial type) on the current sample was 0.83. The final score is the total number of correct answers, and ranges from 0 to 21.

Primary Mental Abilities – Spatial relations – K1 (PMA-K1; Thurstone and Thurstone, 1949; Italian adaptation by Thurstone et al., 1981). This is a measure of spatial visualization ability. It consists of 12 incomplete target figures, each with four different pieces beneath it from which the respondent is asked to choose the one that completes the target figure. The internal consistency is good, the Italian adaptation of the test reportedly achieving an adjusted split-half correlation of *r* = 0.81 in preschoolers (Thurstone et al., 1981). The final score is the total number of correctly chosen pieces, and ranges between 0 and 12.

Working Memory Matrices – sequential and simultaneous – (Lanfranchi et al., 2004, 2015). These are two tasks respectively measuring the sequential and simultaneous aspects of visuo-spatial WM. Both tasks consist of a series of matrices presented on a sheet of paper. The matrices comprise cells measuring 3 cm each. Two trials are presented for each level of difficulty (i.e., the length of the sequence or the positions to learn range from 1 to 4).

In the sequential WM version, matrices of 3 × 3 and 4 × 4 square cells were used. The experimenter showed a path covered on the matrix by a small frog, which jumped onto cells in the matrix, stopping at each cell for 1 s showing sequences moving throughout the matrix, not necessarily in adjacent cells. Participants had to reproduce the sequences of jumps in the right order. In the simultaneous WM version, matrices comprising from 2 × 2 to 4 × 4 square cells were used. The experimenter showed participants the matrix with some cells colored in green and others left blank for 8 s, then showed them an all-blank matrix and asked them to remember the position of the green cells. In both conditions, participants had to respond immediately, and the task was terminated when they failed both trials on the same level of difficulty. In both tasks the final score corresponds to the number of trials completed correctly, and ranges from 0 to 8. The internal consistency is moderately good (0.59 for sequential WM and 0.89 for simultaneous WM, Lanfranchi et al., 2004).

#### Everyday Spatial Activity Questionnaire (ESAQ; Meneghetti et al., 2018)

This is a 6-item questionnaire examining an individual’s ability to move around and reach locations out of doors (e.g., a school, a care center, a public park; e.g., “Can he/she move around the neighborhood unassisted?”), and indoors (e.g., in a classroom, a supermarket; e.g., “At the grocery store, can he/she go and get a product by moving along the aisles?”). It is completed by adults (the parents for the TD children, parents or educators for the individuals with DS), and scored on a 3-point Likert scale (from 1 = very poorly to 3 = very well). If the respondent feels the child shows no evidence of being able to do something, a score of 0 is also allowable. One participant in the DS group had to be excluded from the analysis concerning the ESAQ because some values were missing from the questionnaire. The internal consistency was acceptable: Cronbach’s alpha was.77 (Meneghetti et al., 2018).

### Procedure

Participants were tested individually during two sessions on two different days in the same week (for the participants’ convenience). The first session (lasting around 30 min) was used to administer the Floor Matrix task. The matrix was set up on the floor of a room made available at the day center or school attended by participants. The rooms were similar in size (ranging 4–6 meters in length and width), and enabled the matrix to be aligned with the walls (doors and windows remained visible). The order of presentation of the learning conditions (Observation and Map) was balanced across participants. Each version of the task started with a familiarization phase (two trials) and the instructions emphasized the need for participants to pay careful attention to the sequence of steps shown by the experimenter’s moves or on the map, and then reproduce the same sequence in the right order as best they could.

The second session (lasting around 40 min) was used to administer the measures of individual differences, which were counterbalanced across participants. The tasks were performed in a quiet room (different from the one used for the first session) at the day center or school, where a table and chairs were available. Specific instructions were given for each measure, making sure participants understood the task by practicing with examples before approaching it.

The Everyday Spatial Activity Questionnaire was delivered to parents or guardians after they consented to their child’s participation in the research, and was completed and returned within 2 weeks.

## Results

A Bayesian approach was used for estimations and inferences, mainly because it enables evidence to be quantified taking the uncertainty due to factors not considered into account, including evidence in favor of the null hypothesis, where relevant (McElreath, 2016). The “BayesFactor” (Morey and Rouder, 2015) package and the “brms” package (Bürkner, 2018) of the R software were used for statistical estimation and model fitting.

### Descriptive Statistics

The descriptive statistics (means and standard deviations) of all measures of interest are listed in Table 1, distinguishing between the two groups (DS vs. TD).

**TABLE 1:** Descriptive statistics of individual measures for the two groups.

	Range of possible values	DS group (*N* = 30)		TD group (*N* = 30)	
		*M*	*SD*	*M*	*SD*
Peabody Picture Vocabulary task	0–167	67.20	25.91	69.13	15.62
Raven’s Colored Progressive Matrices	0–36	14.17	5.00	18.80	4.12
Ghost Picture Test	0–21	11.40	3.86	15.87	1.98
Primary Mental Ability, Spatial – K1	0–12	5.30	2.52	7.77	1.85
Sequential working memory task	0–8	3.60	2.27	5.23	1.17
Simultaneous working memory task	0–8	2.77	2.18	4.97	1.35
Floor Matrix task, Map condition	0–7	3.00	1.70	3.53	0.82
Floor Matrix task, Observation condition	0–7	3.50	1.31	4.23	1.19
Everyday Spatial Activity Questionnaire^†^	0–18	9.10	3.92	12.23	2.64

*DS, Down syndrome; TD, typically developing. ^†^For the Everyday Spatial Activity Questionnaire, the DS group has N = 29.*

**FIGURE 2:**
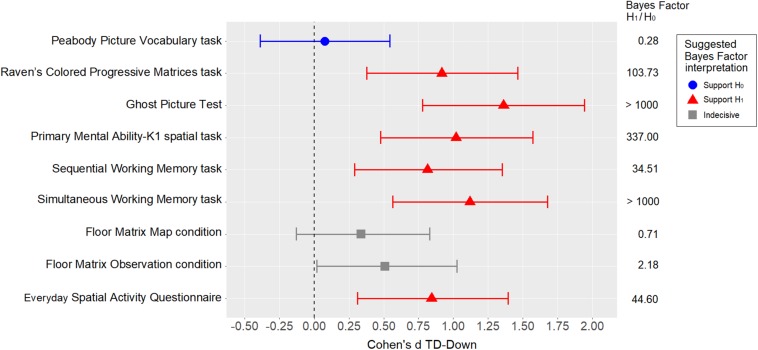
Between-group comparisons using standardized differences (Cohen’s d), with error bars representing 95% BCIs. The Bayes factor refers to *t*-tests.

The standardized difference (Cohen’s d) was used as a measure of the effect size of the between-group comparisons for all measures of interest. Cohen’s d was calculated for each variable of interest using MCMC resampling with the “lmBF” function of the “BayesFactor” package in R. As a measure of uncertainty, 95% Bayesian credible intervals (BCI) were estimated using the percentile method on posterior distributions. In the Bayesian framework, a posterior distribution represents the probability distribution of an effect of interest (e.g., model parameter, standardized difference) after the data has been taken into account, and considering the *a priori* (prior) probability distribution. When objective default priors are used, as in the present case, the posterior distribution is determined solely by the data, and the 95% BCI tends to coincide with the 95% confidence interval reported using the frequentist framework. The standardized differences are shown in Figure 2. Apart from the PPVT-R matching measure, which obviously supports means equality, all the other measures were weaker in the group with DS than in the TD group, with medium to large standardized differences. Interestingly, the differences were smaller in the Floor Matrix task (in both conditions) than in the other visuo-spatial measures.

Given the relatively large number of variables and the relatively small sample size, statistical inference was not a relevant goal at this point. Nonetheless, to obtain an indication of the level of evidence, a t-test Bayes factor (BF) was calculated for each comparison (using the “BayesFactor” package in R). A weakly informed Cauchy prior with rscale = √2/2 was used for H_1_ (set by default by the “ttestBF” function). Referring to Schönbrodt and Wagenmakers (2018), we interpreted a BF > 3 as at least “moderate” evidence of H_1_ (i.e., the hypothesis that the two means are not equal at the population level), and a BF < 1/3 as “moderate” evidence of H_0_ (i.e., the hypothesis that the two means are equal at the population level). Any BF coming between these two cutoffs was regarded as “indecisive” evidence. The BFs and their suggested interpretations are also shown in Figure 2.

### Floor Matrix Task

Linear models were fitted on the Floor Matrix task scores, considered as the dependent variable, to examine the simultaneous roles of group (TD vs. DS), learning condition (Map vs. Observation), and their possible interactions. Because the data consisted of repeated measurements (in the two learning conditions) by participant, mixed-effects linear models were fitted, with random intercepts for the participants. The models were fitted using the “lmBF” function of the Bayes Factor package in R (Morey and Rouder, 2015), which allows for BFs to be computed by comparing the models with vs. without a given effect of interest. Default non-informed priors were used in all models.

**FIGURE 3:**
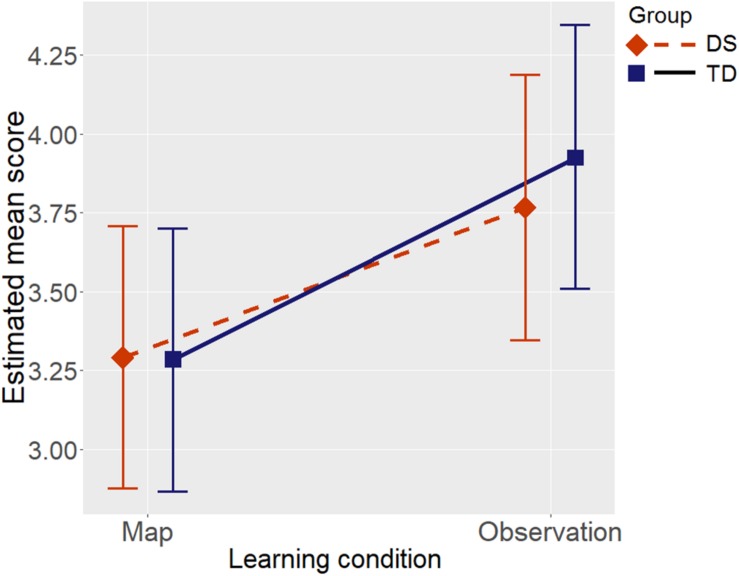
Estimated mean scores in the Floor Matrix task by Group (DS, individuals with Down syndrome; TD, typically-developing children), and by Learning condition, after controlling for Raven’s CPM score. Error bars represent 95% BCIs of the estimated means.

Group showed a main effect, supported by weak evidence, BF = 2.11, when Raven’s CPM was not entered as a covariate in the model; the effect size estimated from the mixed model was medium, with lower scores in the DS group than in the TD group (Cohen’s *d* = -0.55). We opted to insert the effect of Raven’s CPM in the model as a control variable, given that it is a general fluid measure capable of influencing environment learning in individuals with DS as well (Farran et al., 2015; Purser et al., 2015). After controlling for Raven’s CPM the evidence supported no effect of group, BF = 0.30 (H_0_ was suggested). There was a strong effect of the covariate Raven’s CPM on the Floor Matrix task score, BF > 1000.00, so the two groups could be considered as not differing in terms of their scores in the Floor Matrix task once the role of non-verbal fluid reasoning had been taken into account. The learning condition had a main effect supported by fairly strong evidence, BF = 30.60, such that scores were higher in the Observation condition than in the Map condition (see Figure 3; Cohen’s *d* = 0.58). There was evidence against an interaction between group and learning condition, BF = 0.31. Figure 3 shows the estimated score in the Floor Matrix task as a function of group and learning condition: after controlling for Raven’s CPM, the two groups’ performance was much the same, and they both benefited equally from the Observation condition vis-à-vis the Map condition.

### Relations Between Floor Matrix Task Performance and Individual Visuo-Spatial Measures

Additional analyses were run to quantify the relation between visuo-spatial abilities and everyday spatial activity using the Floor Matrix task. All correlations can be found in the online Supporting information (Supplementary Table S1).

#### Visuo-Spatial Abilities

**FIGURE 4:**
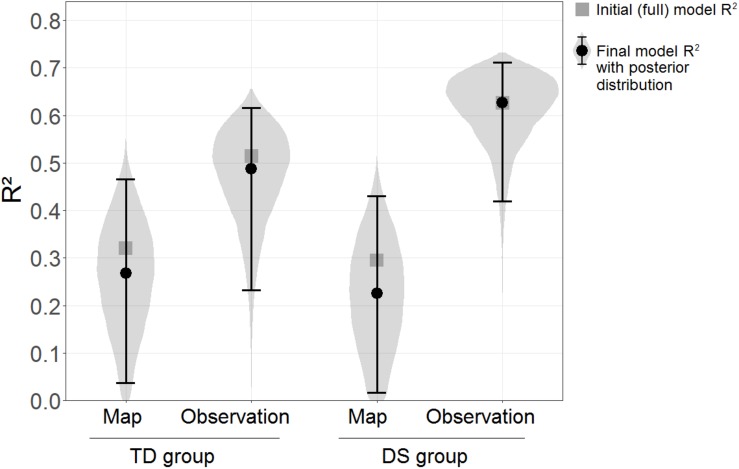
*R*^2^ of the initial models (squares) and final models (circles) with the Floor Matrix task scores as the dependent variables and visuo-spatial abilities as the predictors, estimated separately by Learning condition and Group. Error bars represent 95% BCIs. Violin plots represent posterior distributions of *R*^2^ (i.e., probability distributions of the *R*^2^s having certain values across Learning conditions and Groups).

The Floor Matrix task was treated as the dependent variable in the linear model, and scores in the GPT, PMA-K1, and sequential and simultaneous WM tasks were entered as independent variables. For ease of interpretation, the same model was computed separately for each learning condition and group. The “brm” function of the “brms” package in R was used, which fits Bayesian regression models using the MCMC algorithm implemented in the STAN programming language. Default non-informed priors were adopted for all models, using 4 Markov chains, with 10,000 iterations each, in each model. The outcome of interest was the explained variance, estimated as the model *R*^2^. As shown in Figure 4, the *R*^2^ was clearly higher for the DS group in the Observation condition, *R*^2^ = 0.63, than in the Map condition, *R*^2^ = 0.30. A similar pattern was seen for the TD group, *R*^2^ = 0.51 in the Observation condition vs. *R*^2^ = 0.32 in the Map condition. An evidence ratio was used for comparisons between *R*^2^ in different conditions, calculated as the probability of the *R*^2^ in one condition being superior to the R^2^ in the other (this follows the logic underlying the “hypothesis” function of the “brms” package in R). Although there is no conventional cut-off for the evidence ratio, a value exceeding 39 could be interpreted as roughly equivalent to the amount of evidence given by *p* < 0.05 (two-tailed) in a frequentist framework, as it implies that more than 97.5% of the probability distribution is on one side of a given threshold, and less than 2.5% on the other (0.975/0.025 = 39). The evidence ratio was 134.14 in favor of the *R*^2^ being higher in the Observation condition than in the Map condition in the group with DS, while in the TD group the evidence ratio for the same comparison was only 10.29.

In a second step, a selection procedure was adopted to avoid inflated *R*^2^-values due to irrelevant predictors in the models. The same models as before were fitted, but removing one predictor at a time from each model for as long as this improved its fit. Model fit was assessed using the WAIC and the LOO indexes (Vehtari et al., 2017). The final models included the combinations of predictors that maximized model fit (WAIC and LOO led to the same final models). The final models included as predictors: only PMA-K1 for the TD group in the Map condition; PMA-K1 and simultaneous WM for the TD group in the Observation condition; only simultaneous WM for the DS group in the Map condition; and PMA-K1, and both sequential and simultaneous WM for the DS group in the Observation condition. The *R*^2^ estimated were very similar to those reported above, and the difference between the Observation and Map conditions in the group with DS emerged even more clearly, with an evidence ratio = 306.69.

Figure 4 shows the *R*^2^ of both the initial (full) models and the selected final models, along with the posterior distributions and the 95% BCIs of the *R*^2^ for the final models.

#### Everyday Spatial Activity Questionnaire (Hetero-Assessment)

To better qualify the role of the Floor Matrix task as a measure capturing at least some aspects of everyday spatial activity, the Floor Matrix task scores in the two conditions (Observation and Map) were correlated with the results of the Everyday Spatial Activity Questionnaire (ESAQ).

In the group with DS, performance in the Floor Matrix task correlated strongly with the ESAQ in the Observation condition, *r* = 0.43, while the correlation was negligible in the Map condition, *r* = 0.07. In the TD group, the correlations were negligible in both conditions, *r* = 0.15 (Map) and *r* = 0.00 (Observation). Full details of the correlations between the ESAQ scores and all other variables considered in the present study can be found in Supplementary Table S1.

## Discussion of the Results and Conclusion

The aims of this study were to compare individuals with DS with matched TD children in terms of: (a) their ability to learn increasingly long sequences of steps from actual moves; and (b) how much this learning is supported by their visuo-spatial cognitive abilities and related to their everyday spatial activities.

Concerning the first aim, our results show that – in a vista space setting (with a 4 × 4 matrix of cells placed on the floor of a room) – individuals with DS could learn a path and reproduce it with a sequence of actual moves (turns and straight stretches) in the right order. The two learning conditions considered had a different impact on their performance, however, with the Observation condition proving easier than the Map condition. Intriguingly, this pattern was much the same in the group of TD controls. In fact, after controlling for visuo-spatial reasoning (given its impact on path learning; Farran et al., 2015; Purser et al., 2015), the most relevant result is the difference made by learning condition (in favor of Observation), whereas no group difference emerged.

In particular, in the Observation condition the mean number of steps in the sequences successfully reproduced was around 3–4 (the DS group learnt a mean 3.5 steps, the TD children a mean 4.23). This points to the number of steps learnt in a 4 × 4 floor matrix being higher (descriptively, at least) than when TD children of comparable mental age were administered the WalCT (when they learnt an average of 3 steps). This difference may be attributable to the fact that the squares in the matrix used in the WalCT are placed irregularly on the floor (Piccardi et al., 2014), whereas in our Floor Matrix task they formed a uniform 4 × 4 grid. The number of steps in the sequences learnt by our participants seem more similar to the findings in VE studies in which individuals with DS proved capable of learning and reproducing paths 4 segments long (Courbois et al., 2013; Davis et al., 2014; Farran et al., 2015; Purser et al., 2015; Toffalini et al., 2018), although some of these studies envisaged repeatedly tracing the path until all or most of the segments had been reproduced correctly, not just once as in the present study.

In the Map condition, on the other hand, both of our groups were less successful in reproducing the path: the group with DS learnt a mean 3 steps, children with TD a mean 3.53 steps. As hypothesized for individuals with DS, these results indicate a greater difficulty of using map-based information (simultaneously presenting the whole grid layout on which the path is marked, on a sheet of paper 16 × 16 cm in size) to learn sequences and reproduce them with actual moves in a corresponding grid on the floor (2.3 × 2.3 meters in size). This is consistent with earlier evidence of individuals with DS not benefiting from seeing a map before exploring an environment from a personal point of view (Meneghetti et al., 2017; Toffalini et al., 2018), and their difficulty with applying allocentric information to their actual movements (Lavenex et al., 2015). This difficulty was surprisingly found to apply to TD children too, whereas they might have been expected to benefit from seeing a map before exploring an environment – in the light of previous evidence obtained in preschoolers – (Uttal and Wellman, 1989; Sandberg and Huttenlocher, 2001). Studies on navigation in TD children have differed in some ways, however. For instance, the space tested was limited in the present study (2.30 × 2.30 m in all), whereas previous studies tested children navigating in larger spaces (such as a series of rooms in Uttal and Wellman, 1989; or hallways in Sandberg and Huttenlocher, 2001). Larger spaces can be more useful for detecting the integration of allocentric information (such as room layouts, or walls) with egocentric information (experienced during navigation). In this sense, how TD children benefit from preserving the person’s point of view in learning sequences (indicating the prevalence of egocentric representations) warrants further investigation, because there is evidence in the literature of children 5–6 years old being able to use allocentric information to manage their movements (Nardini et al., 2008, 2009; Ruggiero et al., 2016).

Although these results for Floor Matrix task performance are encouraging, there are some limitations to consider relating to the method used. For a start, the rooms where the matrix was set up contained elements outside the matrix that remained visible to participants, such as doors and windows. This ensured that the task was performed in a “natural” setting, but also gave participants the chance to rely on external reference points as part of their spatial representation, and this would have influenced its final features (e.g., Pennington et al., 2003; Purser et al., 2015). Second, the time of presentation varied in the Observation condition, increasing with the length of the sequence to be remembered, whereas it remained the same in the Map condition (8 s). This difference (involving a generally longer time of presentation in the former condition, for sequences of more than 3 steps at least) could affect performance, and may explain why it was generally better in the Observation condition. It is worth adding that, in a preliminary pilot study, a time of presentation longer than 8 s in the Map condition did not seem beneficial, as it only led to participants’ attention wandering. These methodological aspects need to be carefully considered in further studies.

As for the second aim of our study, to clarify the involvement of visuo-spatial factors in Floor Matrix task performance, our results show how the contribution of individual abilities changed as a function of learning condition (Observation or Map) and group (TD or DS). It is important to note that the individuals with DS were matched with TD children on a verbal measure (receptive vocabulary), but were still weaker than the latter on a series of visual-spatial tasks, both basic sequential and simultaneous WM tasks, and higher-level mental rotation and visualization tasks. These results are not in contrast with the findings of the review by Yang et al. (2014). Individuals with DS performed less well than TD children matched for cognitive functioning (where studies in the review also reported matching them on the PPVT-R) in tasks measuring closure, like our Primary Mental Ability (Spatial – K1) task, which involved identifying the part of a figure needed to complete it. Yang et al. (2014) also reported inconsistent evidence regarding mental rotation, and our results are in line with studies showing a poor performance using a task based on the detection of rotated figures (as in the Ghost Picture Test; in Meneghetti et al., 2018). We also confirmed the poor performance of individuals with DS in simultaneous WM tasks (Carretti and Lanfranchi, 2010), and found that they had difficulty with a sequential WM task as well. This latter result differs from the findings of previous studies (e.g., Lanfranchi et al., 2004), and will need to be confirmed or refuted in future. Overall, the present findings support the assumption that visuo-spatial abilities generally are not a relative strength in individuals with DS (Yang et al., 2014), but this depends on the type of ability tested and the type of measure used. They certainly warrant further investigation in this population.

That said, visuo-spatial abilities influenced Floor Matrix task accuracy in both the individuals with DS and the TD children, to a different degree in the two learning conditions, from Observation or a Map. Judging from our results, the DS group’s visual-spatial abilities (particularly visualization, and sequential and simultaneous WM) were more heavily involved when they learnt a path from direct observation than when they saw a map (only simultaneous WM is involved in the latter case). The same pattern was seen in TD children, but it was weaker (less variance was explained by the model in the Observation condition): visualization and simultaneous WM were especially involved in the Observation condition; and visualization in the Map condition. These results must be considered with caution, however, due to the relatively small sample size of both groups. More specifically, the model selection procedure used to define the “final” best-fitting models or set of predictors should be considered only as an exploratory analysis.

These results prompt some considerations. In the easier learning condition (Observation), visuo-spatial abilities clearly emerged to ensure success in recalling the path, particularly in the group with DS. The contribution of visuo-spatial abilities in the Map condition was less relevant in this group. The same trend was probably at work in the TD group, but with a weaker contribution of their visuo-spatial abilities. Considering the contribution of specific visual-spatial abilities, it seems that a role for visuo-spatial WM (a basic ability) is more detectable in individuals with DS, while the role of visualization (a higher-level ability) seems more apparent in TD children. It is worth noting that the role of sequential WM emerged in the Observation condition for individuals with DS, as expected, but in combination with simultaneous WM (probably due to the sharing of WM processing resources). In the DS population, visuo-spatial WM seems to be a core process in their execution of such a complex cognitive task as path learning. While a higher-level spatial ability like visualization (i.e., the ability to arrange and manage the shapes of objects) seemed relevant in the TD children, this was not the case for mental rotation (unlike previous findings in TD children; Merrill et al., 2016). Other researchers found egocentrically-based abilities (such as the one needed to imagine yourself in different positions in space) related to path learning in TD children (Nazareth et al., 2018).

This seemingly stronger contribution of visuo-spatial abilities in DS than in TD individuals can be explained by the fact that, despite generally weaker visuo-spatial abilities in the DS group than in the TD group (as reported above), some individuals with DS have well-developed visuo-spatial skills. In fact, the DS group showed a greater heterogeneity in its performance, also as regards visuo-spatial skills (see Table 1). There is therefore more room for some individuals with DS – those whose visuo-spatial abilities were relatively strong – to dedicate these resources to underpinning their performance in the Floor Matrix task (especially in the Observation condition, which is generally more manageable for them), whereas those whose underlying abilities are more severely impaired would be unable to do so. This has to do with the question of cognitive profile variability within the same population. There are studies suggesting that the classical profile of individuals with DS does not always apply, and that individual differences in this population can be even twice as great as in the TD population (e.g., Tsao and Kindelberger, 2009; Karmiloff-Smith et al., 2016).

These results confirm the importance of taking the role of individual cognitive abilities into account when examining environment learning in individuals with DS as well (Farran et al., 2015; Purser et al., 2015). At the same time, they offer insight on how to explore the role of visuo-spatial abilities in relation to the variability of task performance in a given population to gain a better picture before drawing any definitive conclusions.

Finally, examining the relations between our participants’ path learning and hetero-assessed everyday life spatial activity (ESAQ) suggested quite a strong association, particularly in the group with DS. This applied especially to this group’s path learning from Observation (*r* = 0.43), rather than from a Map. In the TD group the correlations between Floor Matrix task performance (in both the Map and Observation learning conditions) and the EASQ were negligible. This result supports the use of the Floor Matrix task in individuals with DS to capture aspects of their everyday navigation ability, such as outdoor movements to reach places (as previously suggested by Mitolo et al., 2015). The absence of any relation between Floor Matrix task performance and everyday life spatial activity in the TD group is plausible because 5 and 6 years old children (like those in our TD group) are not required or allowed to go around in the outside world alone (to go to school or visit other parts of their neighborhood). Their parents’ ratings were probably higher than for the DS group because the activities mentioned in the ESAQ were judged as something the children were capable of doing (rather than something they actually did), and there was little or no association between these ratings and the TD children’s Floor Matrix task performance. The adults’ ratings of the individuals with DS are more likely to have captured their real abilities because these individuals were older (from 7.75 to 17.92 years of age), and the older ones would have actual experience of the movements considered. This type of result offers insight on the relationship between everyday experiences of navigation (when hetero-assessed, at least) and an actual navigation task in a controlled setting (as in the Floor Matrix task) in individuals with DS, a relationship that deserves to be better explored. Although these results support the use of the Floor Matrix task to assess large-scale navigation ability with actual moves in a vista space, it would be even better to employ more ecological navigation conditions (such as actual movements in the neighborhood, or to reach a given room in a building) in this population (Yang et al., 2018).

Though further research is certainly needed on the role of small-scale (spatial cognitive) abilities in successful path learning, our results support the spatial cognition model postulating a relationship between small- and large-scale abilities not only in young adults (Hegarty et al., 2006), and TD children (Merrill et al., 2016), but also in cases of atypical development. This relationship can be demonstrated using VE (Farran et al., 2015), and also – as our study newly showed – using actual movements in the environment. Such findings are important not only for the purpose of extending the theoretical framework to cover different populations but also for their various implications. One such implication may be particularly relevant to individuals with DS, for the purpose of training their cognitive abilities (such as visuo-spatial WM) in order to improve other, related cognitive skills (such as spatial learning), or directly practicing with learning from navigation in controlled settings (using the Floor Matrix task, for instance), and analyzing its impact on everyday navigation ability. This issue has yet to be approached directly, but promising evidence has emerged of individuals with DS benefiting from visuo-spatial WM training (Lanfranchi et al., 2017), and future studies can be designed to examine more closely how their navigation abilities might be improved.

Overall, although the results of the present study need to be confirmed, they shed new light on the path learning ability of individuals with DS. They show that: (a) individuals with DS can learn increasingly long sequences of steps in a vista space setting (as in the Floor Matrix task) almost as well as matched TD children, though it seems easier for them to learn from watching a person actually make the moves rather than from looking at a map; and (b) visuo-spatial cognitive abilities are important in supporting path learning accuracy, especially when learning from observing other people’s moves, with visuo-spatial WM seeming particularly relevant in individuals with DS, and visualization ability in TD children. In short, our findings show that individuals with DS are able to learn sequences of steps forming a path from actual moves, and their accuracy in reproducing the path is supported by their individual visuo-spatial abilities.

## Data Availability Statement

The dataset analyzed for this study can be found on Figshare, https://doi.org/doi: 10.6084/m9.figshare.10055438.

## Ethics Statement

The study was approved by the Ethics Committee for Research in Psychology (University of Padua), Number: 2EDFB5F0133B6675FE88D0E3F714B17C. Prior written consent for the children’s participation was obtained from both their parents (or the legal representatives of the individuals with DS).

## Author Contributions

ET organized the database and performed the statistical analysis. CM wrote the first draft of the manuscript. BC, SL, and ET wrote sections of the manuscript. All authors contributed conception and design of the study, manuscript revision, read and approved the submitted version.

## Conflict of Interest

The authors declare that the research was conducted in the absence of any commercial or financial relationships that could be construed as a potential conflict of interest.
