# Supplementary material for: Path Learning in Individuals With Down Syndrome: The Floor Matrix Task and the Role of Individual Visuo-Spatial Measures
Source: Front Hum Neurosci. 2020 Mar 31;14:107. doi: 10.3389/fnhum.2020.00107 (PMC7136450; doi:10.3389/fnhum.2020.00107)
Supplement: Supplementary file 1 [file Table_1.DOCX]

Supplementary Material

**Path learning in in individuals with Down syndrome: the use of flow matrix task and the role of visuo-spatial individual measures**

**Table S1**

Correlations of all measures in the groups of individuals with Down syndrome (below the diagonal, in grey) and typically-developing children (above the diagonal). The sample size is N = 30 for each group.

|  | 1. | 2. | 3. | 4. | 5. | 6. | 7. | 8. | 9. |  |
| --- | --- | --- | --- | --- | --- | --- | --- | --- | --- | --- |
| 1. Peabody Picture Vocabulary task | - | .14 | .28 | .40 | .25 | .25 | .34 | .13 | .11 |  |
| 2. Raven’s Colored Progressive Matrices | .17 | - | .18 | .46 | .06 | .18 | .51 | .27 | .19 |  |
| 3. Ghost Picture Test^1^ | -.15 | .29 | - | .35 | .28 | .11 | .15 | .33 | -.20 |  |
| 4. Primary Mental Ability, Spatial - K1 | .37 | .58 | -.10 | - | .62 | .58 | .52 | .54 | .06 |  |
| 5. Sequential working memory task | .24 | .18 | .46 | .05 | - | .62 | .23 | .58 | .21 |  |
| 6. Simultaneous working memory task | .33 | .41 | .36 | .42 | .65 | - | .33 | .65 | .08 |  |
| 7. Floor Matrix task, Map condition | .29 | .58 | .11 | .34 | .21 | .47 | - | .43 | .15 |  |
| 8. Floor Matrix task, Observation condition | .48 | .39 | .25 | .36 | .70 | .71 | .47 | - | .00 |  |
| 9. Everyday Spatial Activity Questionnaire | .19 | .14 | .50 | -.07 | .72 | .48 | .07 | .43 | - |  |

Note. ^1^Proportion of correct responses.
